# Emerging pathogens: the underestimated risk of *Kodamaea ohmeri* infection in hospitals

**DOI:** 10.3389/fmicb.2025.1572747

**Published:** 2025-05-01

**Authors:** Shuang-Jie Wang, Xia Yu, Jia-hui Liang, Dong-yan Zheng, Cun-Wei Cao

**Affiliations:** ^1^Department of Laboratory, Maternal and Child Health Hospital of Guangxi Zhuang Autonomous Region, Nanning, China; ^2^Joint Inspection Center of Precision Medicine, The People’s Hospital of Guangxi Zhuang Autonomous Region, Guangxi Academy of Medical Sciences, Nanning, China; ^3^Department of Dermatology and Venereology, The First Affiliated Hospital of Guangxi Medical University, Nanning, China; ^4^Guangxi Key Laboratory of Mycosis Research and Prevention, Nanning, China

**Keywords:** *Kodamaea ohmeri*, outbreak, bloodstream infection, neonates, whole-genome sequencing, candidemia, antifungal susceptibility, laboratory diagnostics

## Abstract

**Introduction:**

*Kodamaea ohmeri* is a rare but significant emerging human pathogen, particularly in neonates, with high mortality rates. While most *K. ohmeri* infections are sporadic, they can be underestimated during hospital outbreaks owing to challenges with traditional identification methods. We conducted a retrospective study to determine the diagnostic accuracy of detecting *K. ohmeri* in candidemia.

**Methods:**

Six non-duplicated isolates (initially misidentified as *Candida dubliniensis*) were collected from four patients in a single department over 1 month. Clinical and whole-genome sequencing data of the outbreak strains were evaluated to identify possible outbreaks.

**Results:**

All patients presented atypical features at diagnosis, and isolates had a low minimum inhibitory concentration (MIC) for amphotericin B, 5-fluorocytosine, and echinocandins, except for fluconazole with a high MIC. Notably, Patient 4 had a high MIC for triazoles. The isolates were grouped into three clades based on core genome single-nucleotide polymorphisms and single-copy orthologous genes. Clade 1 contained isolates from Patients 1 and 2, suggesting a common infection source.

**Conclusion:**

This study underscores the need for improved awareness of *K. ohmeri* infections, which, although rare, involve emerging fluconazole-resistant strains. *Kodamaea ohmeri* should be considered a potential nosocomial pathogen capable of causing outbreaks; overlooking these emerging human pathogens may have serious consequences.

## 1 Introduction

*Kodamaea ohmeri*, previously known as *Pichia ohmeri* or *Yamadazyma ohmeri*, has been noted as an emerging fungal pathogen over the last few decades. It is an ascomycetous yeast belonging to the family Saccharomycetaceae and class Ascomycetae. It was first clinically isolated from the pleural fluid of a patient in 1984; however, it was not considered clinically significant (Kregervan Rij, 1984). Subsequently, an immunocompromised patient who developed *K. ohmeri* fungemia died in 1998 (Bergman et al., 1998). Although *K. ohmeri* is an unusual pathogen, it causes various types of infections (Poojary and Sapre, 2009; Al-Sweih et al., 2011; Vivas et al., 2016; Hou, 2019; Yu et al., 2020), particularly in pediatric and neonatal patients, as well as in the older population (Diallo et al., 2019). Most of these cases are sporadic; however, *K. ohmeri* can occasionally cause regional outbreaks (Otag et al., 2005), resulting in severe clinical outcomes. Reports have documented an outbreak of infections in a pediatric intensive care unit in 2005 (Chakrabarti et al., 2014) and a case of bloodstream infection in a premature neonate in 2011 (Liu et al., 2013).

In the present study, we retrospectively analyzed patients with invasive fungal infections. Notably, four cases of *K. ohmeri* infection occurred in the same department within a year. However, we could not determine whether these cases were nosocomial outbreaks. In addition, based on whole-genome sequencing, an outbreak identification determining possible clonal relatedness among the isolates was performed. We aimed to combine whole-genome sequencing data with clinical data for clinicians to make more timely and accurate judgments and provide effective antifungal therapy in patients with infections.

## 2 Materials and methods

### 2.1 Ethical approval

This study was approved by the Ethics Committee of the People’s Hospital of Guangxi Zhuang Autonomous Region (approval number: KY-KJT-2023-49). Written informed consent was obtained from all subjects involved in the study.

### 2.2 Isolates and identification

This study included *K. ohmeri* isolates that were previously identified from the blood of patients admitted to neonatal intensive care units using conventional methods. All fungal isolates were re-identified using polymerase chain reaction (PCR) amplification and internal transcriber spacer 1 (ITS1) and ITS4 sequencing. The clinical data of patients with *K. ohmeri* fungemia were retrieved from the medical record archive.

### 2.3 Antifungal susceptibility testing

The broth microdilution method of the Clinical and Laboratory Standards Institute (CLSI, M60 2^nd^ ed.) was used for testing antifungal susceptibility to nine common antifungal drugs: amphotericin B, itraconazole, 5-flucytosine, caspofungin, fluconazole, voriconazole, anidulafungin, micafungin, and posaconazole. However, no CLSI or European Committee on Antimicrobial Susceptibility Testing (EUCAST) clinical breakpoints or epidemiological cutoff values are available for *K. ohmeri*. The minimum inhibitory concentration (MIC) was evaluated after 24 h of incubation.

### 2.4 Whole-genome sequencing

The genetic relatedness of all *K. ohmeri* isolates was determined using whole-genome sequencing. Total DNA was extracted using a DNA extraction kit, and whole-genome sequencing was performed using the Illumina NovaSeq 6000 platform (Illumina, San Diego, CA, USA), which generated 150-bp paired-end reads. *De-novo* genome assembly was performed using bioinformatics analysis, and final genome assembly was performed. Colonies were identified based on the average nucleotide identity (ANI; Mi et al., 2010). We used Snippy (version 4.6.0) and kSNP3 (version 3.1.2) to align the six core genomes containing all single-nucleotide polymorphisms (SNPs), indels, and deletions. The reference genome was KO1-BLOOD-NICU-MAR2017, which was the earliest genome isolated in the study. The protein sequences were extracted from genome sequence files, and genes common or unique to a genome were analyzed using OrthoFinder (version 2.3.12). Single-copy orthologous genes present in all genomes were used to construct a phylogenetic tree. Single SNPs were based on the core genome shared by all isolates.

## 3 Results

### 3.1 Isolates and identification

**Figure 1:**
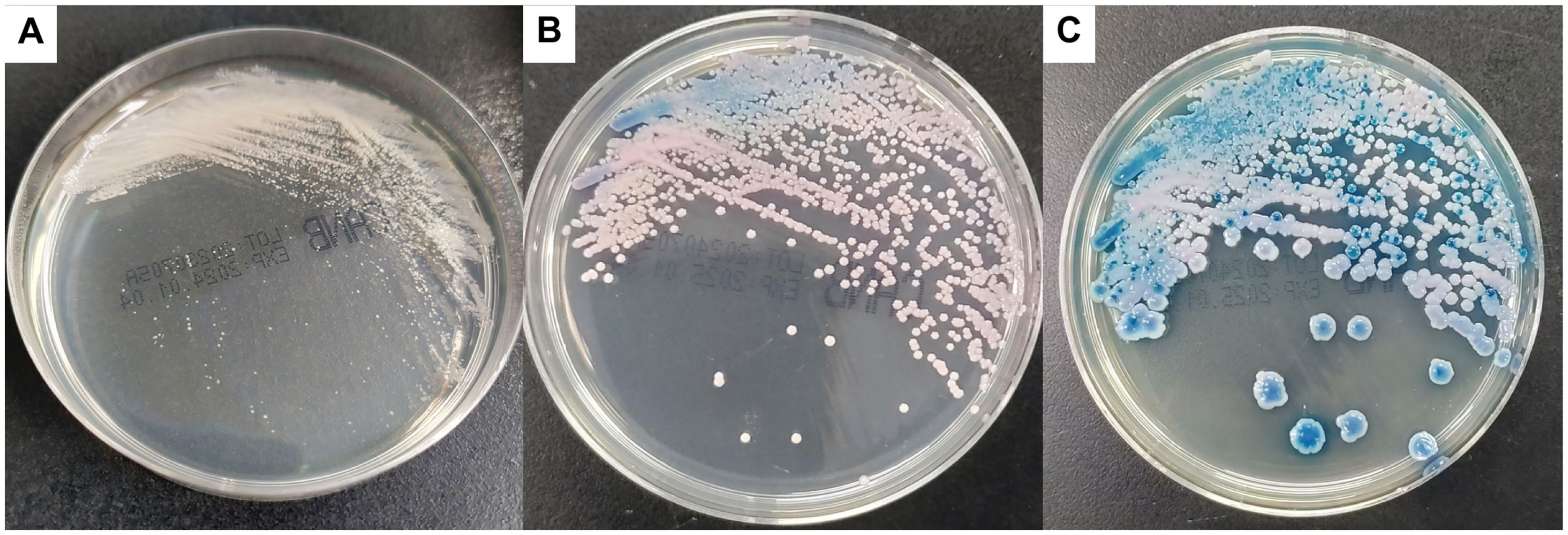
Colonies on CHROMagar Candida culture appeared white at 24 h **(A)**, changed to pink-blue at 48 h **(B)**, and finally turned blue after 5 days **(C)**.

All blood cultures were incubated in an automated culturing system (BioMérieux, Marcy l’Etoile, France). Blood samples in positive flasks were inoculated on Sabouraud dextrose agar at 35°C, and single colonies were subcultured in *Candida* chromogenic plates at 35°C. Catheters and other specimens were inoculated directly into the media. The morphotype on agar plates showed white rugged colonies after 24 h. Subsequently, after 48 h, the colonies were pink-blue and finally changed to blue after 5 days (Figure 1).

**Figure 2:**
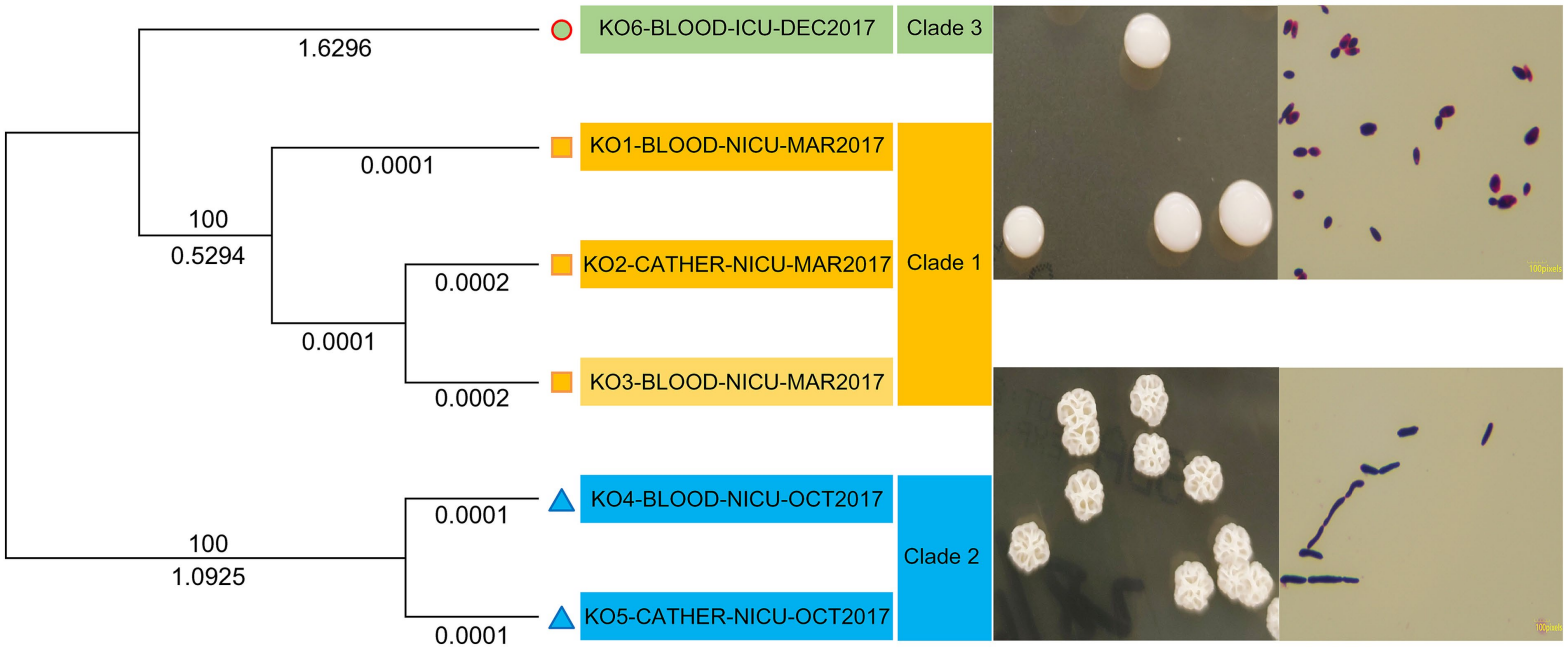
Phylogenetic analysis of six *Kodamaea ohmeri* strains based on core genome SNPs.

All isolates, except for KO4-BLOOD-NICU-OCT2017 and KO5-CATHER-NICU-OCT2017, formed smooth-type colonies, and significant differences were found in spore length compared to the rough-type colonies (KO4-BLOOD-NICU-OCT2017 and KO5-CATHER-NICU-OCT2017; Figure 2). All isolates were initially misidentified as *Candida dubliniensis* using the automatic microbial identification system DL-96II (Zhuhai Dier Biological Co., Ltd., Zhuhai, Guangdong, China). However, different results were obtained for *K. ohmeri* when using a VITEK 2 compact YST card (Biomérieux) with a high identification rate. All *K. ohmeri* isolates were successfully identified based on the basic local alignment search tool (BLAST) results of fungal ITS gene sequencing. The sequencing results showed 100% homology with *K. ohmeri* (GenBank accession number: SKFK01000003.1). Moreover, the isolates were confirmed using whole-genome ANI, and all ANI scores had >99% similarity with *K. ohmeri* (GenBank accession number: GCA_004919595.1).

### 3.2 Patient characteristics

Four patients (three male patients and one female patient; mean age, 28.2 days) developed *K. ohmeri*-related sepsis and were diagnosed with fungal septicemia. Three premature infants with extremely low birth weight (ELBW, <1,000 g) and very low birth weight (VLBW, 1,000–1,499 g) had a mean gestational age of 29.5 weeks. All four patients were treated with broad-spectrum intravenous antibiotics and parenteral nutrition. In addition, all patients had peripherally inserted central catheters (PICCs) or underwent umbilical venous catheterization for venous access when first-positive cultures were obtained. More detailed information is provided in Table 1.

**Table 1:** Infection characteristics of patients with *K. ohmeri* infection.

Isolate no.	Patient 1	Patient 2	Patient 3	Patient 4
	KO1-BLOOD-NICU-MAR2017, KO2-CATHER-NICU-MAR2017	KO3-BLOOD-NICU-MAR2017	KO4-BLOOD-NICU-OCT2017, KO5-CATHER-NICU-OCT2017	KO6-BLOOD-ICU-DEC2017
Sex	Male	Female	Male	Male
Age/Gestational period	20 d/30 + 3 weeks	23 d/30 weeks	26 d/29 weeks	43 d/NA
Birth weight (g)	1,160	1,290	980	3,200
(1,3)-β-d-glucan test (pg/ml)	271.30	168.40	450.9	249.70
WBC (10^9^/L)	9.4	10.1	18.3	9.4
CRP (mg/L)	74.34	149.50	130.43	33.46
Length of stay (d)	76	51	89	67
Source	Blood, venous catheter	Blood	Blood, venous catheter	Blood, venous catheter, gastric fluid, stool
Fever	YES	NO	YES	YES
Antibiotic exposure	Piperacillin/tazobactam, meropenem	Piperacillin/tazobactam, meropenem	Piperacillin/tazobactam, meropenem	Meropenem, metronidazole, vancomycin, imipenem/cilastatin
Risk factors	Intravenous nutrition, Endotracheal intubation, PICC	Intravenous nutrition, Endotracheal intubation, umbilical venous catheterization	Intravenous nutrition, Endotracheal intubation, umbilical venous catheterization, PICC	Intravenous nutrition, Femoral vein catheterization, intestinal obstruction, PICC
Antifungal treatment	Amphotericin B, fluconazole	Fluconazole	Fluconazole, amphotericin B	Fluconazole, voriconazole, amphotericin B
Outcome	Recovered	Recovered	Recovered	Died

CRP, C-reactive protein; WBC, white blood cell; PICC, peripherally inserted central catheter; NA, not available.

### 3.3 Antifungal susceptibility testing

The MICs of the different antifungal agents are listed in Table 2. Nine drugs were tested. The antifungal susceptibilities (MIC ranges) were as follows: amphotericin B, 0.5–1 μg/ml; itraconazole, 0.03–8 μg/ml; 5-flucytosine, 0.06–2 μg/ml; caspofungin, 0.12–0.5 μg/ml; fluconazole, 2–16 μg/ml; voriconazole, 0.06–4 μg/ml; anidulafungin, 0.12–0.25 μg/ml; micafungin, 0.06–0.25 μg/ml; and posaconazole, 0.03–0.12 μg/ml. According to the CLSI recommended breakpoints (in μg/ml) for *Candida* spp., the isolated *K. ohmeri* (KO6-BLOOD-ICU-DEC2017, from Patient 4) was considered resistant to fluconazole, itraconazole, and voriconazole. In three patients (Patients 1, 2, and 3), fungemia was cleared after catheter removal and antifungal therapy (fluconazole or amphotericin B). In contrast, Patient 4 was responsive to amphotericin B therapy, while no response was found to azole agents (fluconazole, itraconazole, and voriconazole) due to higher MIC values. At the final follow-up, the patient had died of severe sepsis and acute necrotizing enterocolitis.

**Table 2:** *In-vitro* antifungal susceptibility data of six *K. ohmeri* isolates.

Antifungal agent	MIC (μg/ml)					
	KO1-BLOOD-NICU-MAR2017	KO2-CATHER-NICU-MAR2017	KO3-BLOOD-NICU-MAR2017	KO4-BLOOD-NICU-OCT2017	KO5-CATHER-NICU-OCT2017	KO6-BLOOD-ICU-DEC2017
Amphotericin B	0.5	0.5	1	1	1	0.5
Itraconazole	0.03	0.03	0.12	0.03	0.12	8
5-Fluorocytosine	1	1	2	1	1	≤0.06
Caspofungin	0.12	0.25	0.5	0.5	0.25	0.12
Fluconazole	2	2	2	2	2	16
Voriconazole	0.12	0.06	0.12	0.12	0.12	4
Anidulafungin	0.12	0.25	0.25	0.25	0.25	0.12
Micafungin	0.06	0.25	0.12	0.25	0.12	0.25
Posaconazole	0.03	0.03	0.12	0.06	0.06	0.12

MIC, minimum inhibitory concentration.

### 3.4 Molecular characterization of *Kodamaea Ohmeri* isolates

**Figure 3:**
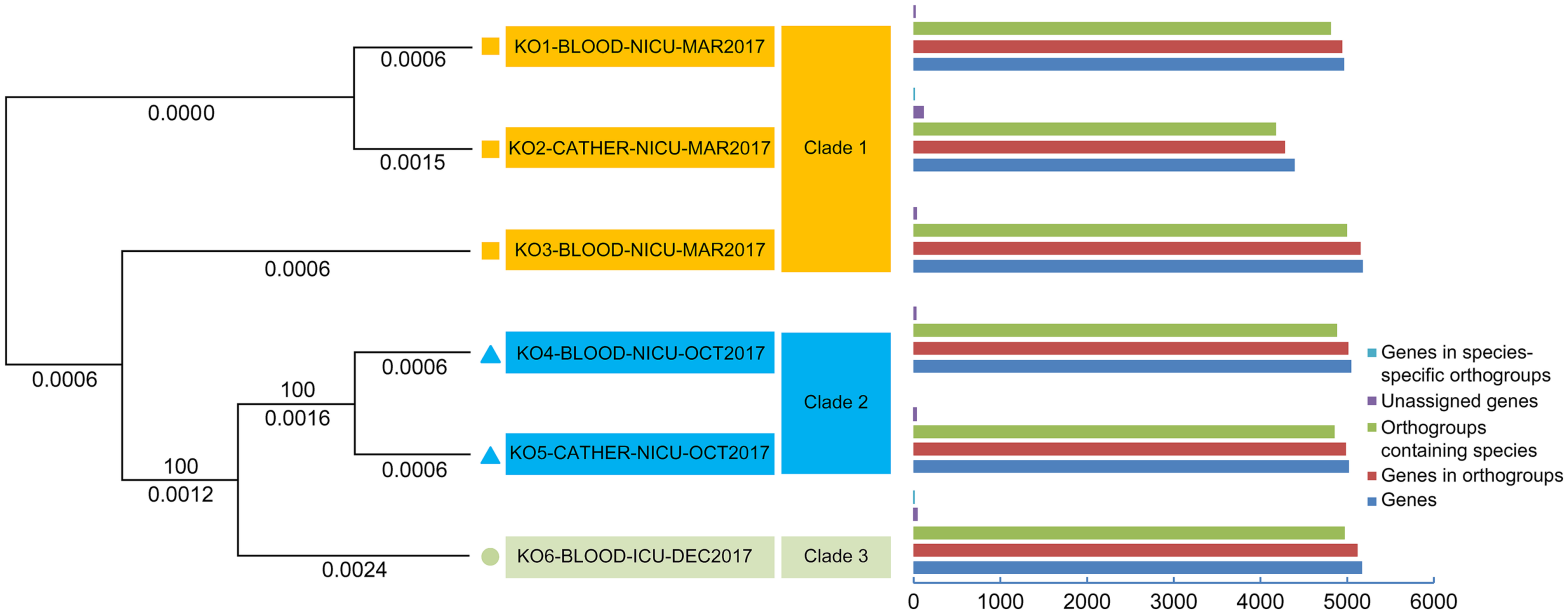
Phylogenetic analysis of six *Kodamaea ohmeri* strains based on single-copy orthologous genes.

Table 3 indicates that the average number of total bases of the six isolated *K. ohmeri* strains was 12,351,080.17 (range: 12,314,056–12,459,599), and the GC% was 42.71% (range: 42.69–42.72%), consistent with those of other published *K. ohmeri* genomes (Chew et al., 2022). The genome ORF number was 4,968.17 (range: 4,404–5,194). The RNA prediction results showed that the tRNA number was 290.5 (range: 280–312), and the rRNA number was 4.67 (range: 2–10). The number of proteins was also predicted for each of the six strains. The number of genes in the orthogroups ranged from 4,292 to 5,155. The number of unassigned genes ranged from 24 to 112, that of orthogroups containing species ranged from 4,176 to 5,003, and that of genes in species-specific orthogroups ranged from 0 to 19. Details are presented in Figure 3.

**Table 3:** Basic genomic information of six *K. ohmeri* isolates.

Isolate no.	Total bases (bp)	G + C (%)	Gene ORF no.	tRNAs no.	rRNAs no.
KO1-BLOOD-NICU-MAR2017	12,328,393	42.692	4,970	289	2
KO2-CATHER-NICU-MAR2017	12,459,599	42.719	4,404	312	10
KO3-BLOOD-NICU-MAR2017	12,350,776	42.713	5,194	288	3
KO4-BLOOD-NICU-OCT2017	12,314,056	42.713	5,045	287	5
KO5-CATHER-NICU-OCT2017	12,320,175	42.71	5,023	280	3
KO6-BLOOD-ICU-DEC2017	12,333,482	42.713	5,173	287	5
Average	12,351,080.17	42.71	4,968.17	290.5	4.67

ORF, open reading frame.

### 3.5 Phylogenetic analysis of *Kodamaea Ohmeri* isolates based on core genome SNPs and single-copy orthologous genes

All sequences were aligned against the reference (KO1-BLOOD-NICU-MAR2017) for SNP and insertion/deletion variant identification (Table 4). The phylogenetic tree based on the core genome SNPs of the six isolates showed that they could be divided into three clades: clades 1, 2, and 3. However, they did not belong to the same cluster in the tree (Figure 2). Three strains (KO1-BLOOD-NICU-MAR2017, KO2-CATHER-NICU-MAR2017, and KO3-BLOOD-NICU-MAR2017) from Patients 1 and 2 clustered on the same sub-branch that belonged to clade 1 and were genetically indistinguishable, with a few SNP differences in the genome, suggesting that they were either transmitted from one patient to the other or transmitted to both patients from a common but unidentified source. The isolate of KO4-BLOOD-NICU-OCT2017 was closely related to the strain KO5-CATHER-NICU-OCT2017 from Patient 3, which belonged to clade 2. However, one isolate, KO6-BLOOD-ICU-DEC2017, from Patient 4 was unclustered and belonged to clade 3.

**Table 4:** Comparison of variants among the isolates (using the isolate KO1-BLOOD-NICU-MAR2017 as the reference).

Isolate no.	COMPLEX	DEL	INS	MNP	SNP	Total
KO2-CATHER-NICU-MAR2017	9	0	4	3	23	39
KO3-BLOOD-NICU-MAR2017	8	0	4	4	27	43
KO4-BLOOD-NICU-OCT2017	3,555	1744	1,535	706	38,309	45,849
KO5-CATHER-NICU-OCT2017	3,641	1755	1,571	616	38,348	45,931
KO6-BLOOD-ICU-DEC2017	4,449	2046	1900	913	45,565	54,873

DEL, deletion; INS, insertion; SNP, single-nucleotide polymorphism; MNP, multiple nucleotide polymorphism.

In total, 3,669 single-copy orthologous genes were selected for phylogenetic analysis (Figure 3). The isolates were classified into three clades (clades 1, 2, and 3). The strains (KO1-BLOOD-NICU-MAR2017, KO2-CATHER-NICU-MAR2017, and KO3-BLOOD-NICU-MAR2017) were also clustered into one branch (clade 1). Two strains (KO4-BLOOD-NICU-OCT2017 and KO5-CATHER-NICU-OCT2017) exhibited clear clustering trends (clade 2). Only one isolate (KO6-BLOOD-ICU-DEC2017) was unique, and it formed a separate cluster (clade 3).

## 4 Discussion

Historically, *K. ohmeri* was widely used in the fermentation of fruits, pickles, and rinds in the food industry. It is considered an uncommon opportunistic fungus, and human infections are rare. However, cases of *K. ohmeri* infection have increased in recent years (Das et al., 2015), particularly in immunocompromised patients, older individuals, and neonates, with a mortality rate of up to 50% (Zhou et al., 2021). In the present study, we found that *K. ohmeri* has emerged as a potential human pathogen capable of causing nosocomial infections, sporadic infections, and outbreaks. We also noted the presence of fluconazole-resistant strains. Therefore, emerging fungal infectious diseases, especially invasive ones, are serious problems that should not be ignored.

*Kodamaea ohmeri* usually causes invasive infections, including fungemia, endocarditis, and skin infections. However, *K. ohmeri* is often misidentified as a *Candida* sp. based on chromogenic *Candida* agar tests or phenotypic characteristics (Lee et al., 2007; Zhou et al., 2019). Hence, accurate identification of less common fungi is important. Polyphasic approaches, including VITEK identification, MALDI-TOF MS, and molecular biology, can be used to identify *K. ohmeri* quickly and accurately (Distasi et al., 2015; Kanno et al., 2017; Hou, 2019). The clinical manifestations of neonatal *K. ohmeri* infection lack specificity, and clinical characteristics may differ among age groups, which complicates early diagnosis. Blood testing for 1,3-β-D-glucan (BDG), a non-culture-based diagnostic test, can expedite the diagnosis of candidemia and has been used as a guide to shorten the time to initiation of antifungal therapy (De Pascale et al., 2020). In this study, four patients were tested for blood BDG, and all tested positive, suggesting that BDG is valuable in diagnosing candidemia caused by *K. ohmeri*. Laboratory test results also showed that the white blood cell count and CRP in these patients increased at varying degrees. Upon entering the bloodstream, *K. ohmeri* cells are engulfed by phagocytes and expose BDG. A previous study (Cabanillas Stanchi et al., 2019) found increased CRP levels in children with fungal infection. The combined detection of WBC, CRP, and BDG can increase the early diagnostic accuracy of *K. ohmeri* infections, so as to provide the basis for clinical treatment. Fever is the most common clinical manifestation in patients with *K. ohmeri* infection (Zhou et al., 2021). In addition, patients exhibit cough, expectoration, and breathing difficulty. In some cases, patients show hematological disorders, as well as digestive tract and central neurological symptoms.

According to the literature (Han et al., 2004; Benjamin et al., 2010; Yamamoto et al., 2013; Santolaya et al., 2014; Bassetti et al., 2020), risk factors for invasive fungal infections, including malignancy, immunosuppression, chemotherapy, exposure to broad-spectrum antibiotics, central venous catheterization, intravenous drug use, endotracheal intubation and mechanical ventilation, respiratory disease, and parenteral nutrition, are highly prevalent in patients with *K. ohmeri* fungemia. However, the risk factors for neonates may differ, especially in premature neonates admitted to the ICU with ELBW or VLBW (Bennett, 2017). Three-quarters of the patients in this study had both ELBW and VLBW, and all were premature births. They also had longer ICU stays, with a mean hospital stay of 70 days. Therefore, it is important to identify the risk factors for *K. ohmeri* infections, particularly in neonates.

There are currently no uniform treatment guidelines for *K. ohmeri* infections, resulting in the use of diverse treatment regimens. The main treatment measures for fungemia include the removal of central venous catheters and antifungal therapy. Many infections caused by *K. ohmeri* are effectively treated with azoles, amphotericin B, and echinocandins. Successful treatment with fluconazole has also been reported (Chakrabarti et al., 2014). Similarly, in the present study, patients were successfully cured using fluconazole or amphotericin B (except for Patient 4). However, fluconazole-resistant *K. ohmeri* strains have been reported (Yang et al., 2009). Notably, we found in this study especially for Patient 4, that the MIC levels for antifungal triazole agents (fluconazole, itraconazole, and voriconazole) tended to be higher than those for other antifungal agents. Initially, Patient 4 was consecutively treated with fluconazole and voriconazole; however, the persistence of fungemia led to a change in treatment to amphotericin B, and fungal cells were cleared in later stages. Therefore, in clinical practice, when selecting antifungal agents for treatment of *K. ohmeri* infections, attention should be paid to the results of drug sensitivity tests and the use of drugs adjusted over time.

The global incidence of *K. ohmeri* infections has exhibited a clear upward trend, particularly in Asia, with sporadic cases and regional outbreaks. An Indian tertiary care center presented the largest cluster of *K. ohmeri* fungemia, with 15 deaths from a single center (Chakrabarti et al., 2014). Researchers re-identified 38 isolates of *K. ohmeri* that were misidentified as *Candida tropicalis* based on phenotypic characteristics. Using molecular techniques, such as fluorescent amplified fragment length polymorphism, all *K. ohmeri* isolates were found to have >92% similarity, and the majority (63.2%) of the isolates had a possible clonal origin with >96% similarity. Similar observations have been reported from China. There were six cases of *K. ohmeri* infections and no deaths in a retrospective analytical study in 2010 (Liu et al., 2013). All strains identified as *K. ohmeri* were subjected to randomly amplified polymorphic DNA (RAPD) analysis to explore their epidemiological characteristics. The same genotype was marked by RAPD fingerprinting results, suggesting that these strains belonged to the same clone and were transmissible within the hospital. All adverse events caused by *K. ohmeri* indicated that it has the potential to cause outbreaks and can have serious consequences in hospitals.

Notably, in our study, *K. ohmeri* infections occurred in the same year. However, we were unable to determine whether an outbreak had occurred. Phylogenetic analysis was performed to analyze the differences between *K. ohmeri* intraspecies genomes. Two phylogenetic trees were constructed, one based on single-copy orthologous genes and the other based on core genome SNP profiles. Both analytical approaches showed the same clustering pattern for the isolates, which were grouped into three distinct clades (clades 1, 2, and 3). This finding indicates a high variation among the *K. ohmeri* isolates. All cases occurred sporadically, with no outbreaks or clusters of infections. However, it should be noted that certain isolates (from Patient 1) exhibited strong genetic similarities with other isolates (from Patient 2) and were temporally and geographically related. Therefore, it is likely that the patients with these isolates were infected via patient-to-patient transfer or through acquisition from a common source. Additional investigation is required to accumulate a larger number of cases and validate these findings.

In summary, *K. ohmeri* can act as a rare opportunistic pathogen, causing hospital-acquired fungemia in the preterm population and potentially causing nosocomial outbreaks. Molecular epidemiology can rapidly identify outbreak clusters and provide a basis for the precise prevention and control of nosocomial infections. Emerging fluconazole-resistant strains, which commonly burden hospitals, are of concern.

## Data Availability

The data presented in the study are deposited in the BioSample repository (accession numbers SAMN48048701, SAMN48048702, SAMN48048703, SAMN48048704, SAMN48048705, and SAMN48048706) and Sequence Read Archive (SRA) repository (accession number PRJNA1252389).
